# Role of Gut-Related Peptides and Other Hormones in the Amelioration of Type 2 Diabetes after Roux-en-Y Gastric Bypass Surgery

**DOI:** 10.5402/2012/504756

**Published:** 2012-05-07

**Authors:** Mirella P. Hage, Bassem Safadi, Ibrahim Salti, Mona Nasrallah

**Affiliations:** ^1^Division of Endocrinology, Department of Internal Medicine, American University of Beirut-Medical Center, P.O. Box 11-0236/D23 Riad El-Solh, Beirut 1107 2020, Lebanon; ^2^Department of Surgery, American University of Beirut-Medical Center, P.O. Box 11-0236/D23, Riad El-Solh, Beirut 1107 2020, Lebanon

## Abstract

Bariatric surgery is currently the most effective and durable therapy for obesity. Roux-en-Y gastric bypass surgery, the most commonly performed procedure worldwide, causes substantial weight loss and improvement in several comorbidities associated with obesity, especially type 2 diabetes. Several mechanisms are proposed to explain the improvement in glucose metabolism after RYGB surgery: the caloric restriction and weight loss per se, the improvement in insulin resistance and beta cell function, and finally the alterations in the various gastrointestinal hormones and adipokines that have been shown to play an important role in glucose homeostasis. However, the timing, exact changes of these hormones, and the relative importance of these changes in the metabolic improvement postbariatric surgery remain to be further clarified. This paper reviews the various changes post-RYGB in adipokines and gut peptides in subjects with T2D.

## 1. Introduction

The epidemic of obesity continues to increase, followed in close parallel by T2D, and the World Health Organization estimates show that by 2015, around 2.3 billion adults will be overweight and greater than 700 million will be obese [1]. Recommendations to achieve weight loss include primarily lifestyle measures such as dietary therapy and exercise, limited pharmacological treatment, and bariatric surgery. Bariatric surgery has proven so far to be the most effective and durable treatment option for both the excess weight and the related comorbidities [2, 3]. Strong evidence has revealed that in addition to inducing major weight loss, bariatric surgery further ameliorates diabetes, hypertension, and dyslipidemia [4]. Of those with T2D, 78% had complete resolution following surgery and diabetes improved or resolved in 86.6% of patients. The greatest effect on weight loss and diabetes resolution was seen in patients undergoing biliopancreatic diversion/duodenal switch followed by gastric bypass and then banding procedures [5].

**Figure 1:**
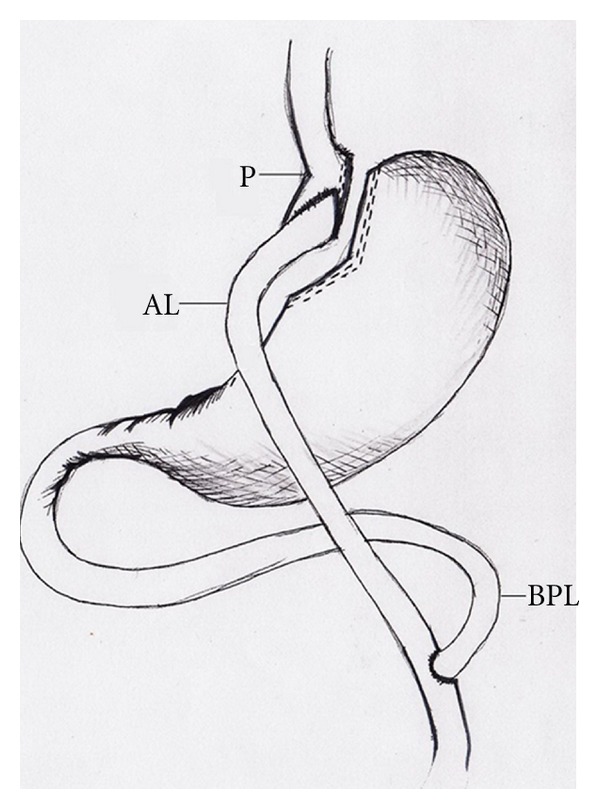
Roux-en-Y gastric bypass. P: gastric pouch. AL: alimentary limb. BPL: biliopancreatic limb.

Among the various techniques in bariatric surgery, RYGB is the most common bariatric surgery performed worldwide and is considered by many surgeons as the “gold standard” procedure [6]. The RYGB operation was developed in the 1960's following observations of weight loss after gastric resection for peptic ulcer disease. Surgeons worked on multiple alterations of the operation and deduced that for effective weight reduction, the stomach size needs to be reduced to less than 50 mLs. This small part of the stomach that remains in continuity with the digestive tract is referred to as the gastric pouch, whereas the majority of the stomach and the duodenum are excluded and are no longer in direct contact with food. The gastric pouch is then reattached to the small intestines using either staples or sutures, and this connection is referred to as the stoma. The preferred way to connect the pouch to the small intestine is via a Roux-y-configuration as shown in Figure 1. In the RYGB, the food goes across the pouch into the “alimentary limb”, whereas the biliary and pancreatic juices flow a distance away from the pouch to form what is referred to as the “biliopancreatic limb” to minimize the harmful effects of “bile reflux” [7].

Several studies have demonstrated the dramatic effect of RYGB on T2D occurring as early as 6 days postoperatively long before major weight loss has occurred [8]. Elucidating the mechanisms of improvement of diabetes after RYGB may lead to a better understanding of the pathophysiology of T2D and guide the search for novel therapies. Hypothesis linking the early and rapid metabolic improvement to bariatric surgery have focused on hormonal changes, namely, adipokines and gut peptides. Therefore, the purpose of this paper is to critically review the recent data and clinical studies addressing the changes in gut-related peptides and other hormones after RYGB surgery and the resulting alterations in metabolic profile.

## 2. Literature Search

A Pubmed search through the English Literature was conducted from 1979 to 2010 using various combinations of the following key words: “adiponectin”, “amylin”, “bariatric surgery”, “gastric bypass”, “gastrointestinal hormones”, “GLP-1”, “ghrelin”, “gut hormones”, “insulin”, “leptin”, “metabolic surgery”, “obesity”, “oxyntomodulin”, “peptide YY” (PYY), and “Roux-en-Y gastric bypass” (RYGB). Only longitudinal and cross-sectional studies assessing hormonal changes after RYGB surgery in obesity and diabetes from year 2000 to 2010 were identified and included due to paucity of studies addressing this issue before year 2000.

## 3. Mechanisms of Improvement of Diabetes after RYGB Surgery

Weight loss per se and the decrease in fat mass induced by bariatric surgery reduce insulin resistance through the direct and indirect effects of adipocytokines and through the fall in lipid content in both liver and muscle. Furthermore, caloric restriction imposed by bariatric surgery allows the beta-cells to rest and they are thus minimally challenged. A significant improvement in glucose homeostasis that is independent of weight loss can be achieved by following a very low-caloric diet [9, 10]. One study by Henry et al. showed that a 330 cal/day diet resulted in reduction in fasting plasma glucose from 297 mg/dL to 138 mg/dL over 40 days with 87% of this drop occurring in the first 10 days [10]. However, the effects of weight reduction and caloric restriction alone do not account for the dramatic and sustained effects of bariatric surgery on diabetes, long after negative caloric balance had ceased. Two hypotheses have been proposed to explain how bariatric surgery improves diabetes early on.

1. Hind gut hypothesis: this hypothesis holds that diabetes control results from the rapid delivery of nutrients to the lower intestine overstimulating the L cells to release gut hormones such as GLP-1, peptide YY, and oxyntomodulin. These hormones exert anorectic and insulinotropic effects to various extents thus improving glucose homeostasis [11, 12].
2. Foregut hypothesis: in normal conditions, the passage of nutrients along the proximal bowel stimulates the production of an unidentified factor with anti-incretin properties responsible for insulin resistance and abnormal glycemic control. Thus, the exclusion of the proximal intestine would reduce the production of anti-incretins and would therefore increase insulin secretion and action and improve diabetes [12–14].

Rubino et al. supported the foregut hypothesis as an explanation for the improvement in glucose homeostasis after RYGB surgery. They showed that excluding the proximal intestine in Goto-Kakizaki (GK) diabetic rats that have undergone gastrojejunostomy ameliorated their diabetes compared to rats with an intact duodenal passage. Conversely, in rats that had undergone duodenal-jejunal bypass, restoration of their duodenal passage reestablished their impaired glucose tolerance [15]. A recent study by Knop suggested a possible role for glucagon or gut-derived glucagonotropic signaling as diabetogenic signal of the foregut hypothesis [14].

Since RYGB significantly changes the anatomy of the gastrointestinal tract, alteration in the secretion of several gut peptides ensues. These hormones are involved in appetite regulation and energy balance and have been implicated in glucose homeostasis as well. In the next section follows a detailed review of individual peptides and hormones.

## 4. Gut Hormones

### 4.1. Ghrelin

Ghrelin is a 28 aminoacid peptide secreted predominantly by the X/A-like enteroendocrine cells of the fundus of the stomach [16]. Plasma ghrelin levels rise nearly twofold before a meal and fall within one hour after eating [17]. It is the only known circulating orexigen. Ghrelin circulates in two different forms: acylated ghrelin and desacylated ghrelin [18]. Acyl-ghrelin accounts for less than 10% of the total circulating ghrelin. It binds to GHSR1a receptor and stimulates food intake as well as GH secretion [19]. Des-acyl ghrelin, the major circulating form of ghrelin, does not bind to GHSR1a receptor but it is not biologically inactive. However, it has been shown to counteract the effects of acyl-ghrelin on insulin secretion and glucose metabolism in humans [20] and reduce food intake in mice [21].

*How does ghrelin contribute to diabetes resolution after RYGB surgery?*

Ghrelin has been shown to increase levels of GH [22], cortisol, and epinephrine [23], three counter regulatory hormones that oppose insulin action. It decreases as well secretion of the insulin sensitizing hormone adiponectin [24]. In addition, ghrelin suppresses intracellular insulin signaling in cultured hepatocytes [25] impairing hepatic insulin sensitivity. Furthermore, ghrelin could influence insulin secretion by a direct effect on the pancreas as the ghrelin receptor GHSR1-a is expressed in various tissues including the pancreatic islets [26, 27]. Exogenous ghrelin administration decreases insulin secretion in both human and animal studies [28, 29]. Some studies report improved glucose disposal by muscle under ghrelin; however, the effect of ghrelin on the liver is that of insulin insensitivity so that the overall riding effect is that of an increase in plasma glucose [30, 31]. Therefore suppression of ghrelin after RYGB surgery is associated with improved glucose homeostasis.

Studies reporting changes in ghrelin levels after RYGB surgery have shown conflicting results (see Table 1). Ghrelin levels have been reported to decrease as early as during the intraoperative period following division of the stomach [32] or later [33–38]. Other studies have reported no change in ghrelin levels after RYGB surgery [34, 39–41].

### 4.2. Glucagonlike Peptide-1 (GLP-1)

Glucagonlike peptide-1 is a 30 aminoacid peptide secreted by the L cells of the distal ileum and colon in response to ingested nutrients. It enhances glucose-dependent insulin release and improves beta cell function [42, 43]. Furthermore, it inhibits glucagon secretion, delays gastric emptying and indirectly decreases food intake [44]. Circulating GLP-1 has a short half-life of less than 2 minutes principally due to its inactivation by the plasma enzyme dipeptidyl peptidase-IV (DPP-IV) [45]. Chronic subcutaneous GLP-1 administration improved glycemic control and decreased body weight in type 2 diabetic patients [46]. In fact, GLP-1 R agonists, resistant to DPPIV inactivation, have been successfully used in the treatment of diabetes. Similarly, direct DPPIV inhibitors also improve glycemic control in T2D, although to a lesser extent, likely because endogenous GLP-1 levels are low in diabetes. Therefore, GLP-1 retains its insulinotropic property in diabetic subjects, but its circulating levels are decreased [47, 48].

In theory, GLP-1 should increase after RYGB due to the rapid nutrient delivery to the ileum where most of the L cells are located. Most data obtained regarding changes in GLP-1 after RYGB surgery have shown an increase [49–58], supportive of this theory, except for one study which showed a decrease in both controls and subjects [59]. Few others have shown no change [41, 60, 61]. A summary of the studies is presented in Table 2.

### 4.3. Glucose-Dependent Insulinotropic Polypeptide (GIP)

Glucose-dependent insulinotropic peptide formerly known as gastric inhibitory polypeptide is a 42 aminoacid peptide that is secreted by the K cells of the duodenum and jejunum in response to ingested nutrients. It enhances glucose-dependent insulin secretion [62] and promotes beta cell proliferation [63]. Higher levels of basal GIP as well as an increased early phase postprandial GIP response were seen in obese subjects compared to lean individuals [48]. In subjects with T2D, the overall effect of GIP seems to be in favor of hyperglycemia. In a recent study by Chia et al., exogenous administration of GIP raised glucose levels in type 2 diabetic patients in both early and late postprandial phases [64]. One mechanism is the impaired insulinotropic action of GIP which has been observed particularly during the late phase of insulin secretion [47]. This could be explained by a defective expression of GIP receptors as observed in Zucker diabetic fatty rats [65]. Secondly, although GIP is an insulinotropic hormone, an elevation of glucagon secretion with GIP infusion was observed in the early postprandial phase counteracting insulin glucose lowering effect. Thirdly, exogenous administration of GIP, as reported by Chia et al., worsened hyperglycemia in the late postprandial phase evoking a potential suppressive effect of GIP on GLP-1 [64]. Furthermore, GIP may be directly implicated in fat metabolism and development of obesity by influencing insulin sensitivity of adipocytes. GIP promotes deposition of fat in adipose tissues and inhibits lipolysis [66, 67]. Mice lacking GIP receptors [68] or K cells [69]were protected from obesity when fed a high-fat diet, and young prediabetic *ob/ob* mice treated with (Pro^3^) GIP a specific and stable GIP receptor antagonist prevented the development of diabetes and related metabolic abnormalities in these rodents [70] Therefore, GIP receptor antagonists, by opposing GIP's anabolic action on adipose tissue, could represent a new treatment modality for obesity [71].

Since GIP is secreted by the proximal gut, bypassing the duodenum and part of the jejunum in the RYGB surgery is expected to result in a decrease in GIP secretion and therefore a more favorable glycemic milieu. Studies involving GIP and RYGB surgery have shown inconsistent results. Reduced levels postoperatively have been reported in some studies [60, 61] while others reported no change or an increase in GIP levels after surgery as shown in Table 3 [49, 50, 72, 73].

### 4.4. Oxyntomodulin (OXM)

Oxyntomodulin is a 37 aminoacid peptide derived from proglucagon cosecreted with GLP-1 and PYY from the L cells of the distal gut in response to ingested nutrients [74]. Central and peripheral administration of OXM has been observed to reduce food intake in rats [75]. Infusion of OXM in humans prolonged gastric emptying, reduced pancreatic exocrine secretion, and decreased food intake [76, 77]. Furthermore, subcutaneous administration of OXM decreased body weight in overweight and obese individuals [78]. However, the effect of exogenous OXM on glycemic control in diabetic subjects has not been assessed.

Similar to GLP-1 and PYY, bariatric surgeries that accelerate the delivery of enteral nutrients to distal intestine should result in an increase in OXM levels. One recent study by Laferrère et al. observed a marked increase in OXM levels 1 month after RYGB surgery in morbidly obese women with T2D [79].

### 4.5. Peptide YY (PYY)

Peptide YY is a 36 aminoacid peptide, member of the pancreatic polypeptide family, secreted by the L cells of the terminal ileum and colon postprandially in response to food [80]. It circulates in two endogenous forms: PYY(1–36) and PYY(3–36) with the latter constituting the main circulating form [81]. PYY(3–36) binds to the neuropeptide Y subtype 2 receptor (NPY2) in the hypothalamus and decreases appetite and food intake as seen in rodents and humans when infused peripherally [82, 83]. Chronic intravenous administration of PYY in obese rodents resulted in a dose-dependent weight reduction [84]. PYY(3–36) infusion also decreased ghrelin levels [83]. Furthermore, when a selective NPY2 polyethylene glycol-conjugated (PEGylated) peptide agonist was infused in diet-induced obese (DIO) mice, improvements in glucose disposal as well as in plasma insulin and glucose levels were observed [85].

Similar to GLP-1, PYY levels are low in obesity [86, 87] and at least a blunted response to a meal has been described in T2D. Levels increase after RYGB surgery in both obese and diabetic subjects and may account for the added beneficial satiety and metabolic effects of this procedure [35, 39, 40, 51, 56, 57, 59, 88]. A summary of the studies is presented in Table S1 (see Table S1 in supplementary materials available online at doi:10.5402/2012/504756). 

### 4.6. Amylin

Amylin is a 37 aminoacid peptide cosecreted with insulin from pancreatic beta cells. It plays a role in glucose homeostasis by slowing gastric emptying [89], suppressing glucagon postprandial secretion and decreasing food intake [90, 91]. Furthermore, amylin has been found to have a synergistic effect with leptin on eating, body weight, and body adiposity and a synergistic effect with PYY in controlling food intake [92, 93]. A state of amylin deficiency has been observed in diabetes as seen in rats with streptozotocin-induced B-cell damage and in the spontaneously diabetic BB Wistar rats [94] as well as in humans [95]. Therefore, as with insulin, secretion of amylin requires the presence of functioning beta cells. In parallel to the insulin levels, fasting plasma amylin levels are increased in patients with early type T2D and obesity, suggestive of a state of amylin resistance [96]. Consequently, high amylin levels are expected in obese subjects with T2D, and a reduction in their levels with weight loss should be observed in theory.

Studies supportive have shown that male Sprague Dawley obese rats had a significant reduction in plasma amylin levels after RYGB surgery [97]. Similarly, a decrease in amylin levels was reported by Bose et al. in morbidly obese diabetic women at one month after RYGB surgery [98]. Kashyap et al., however, reported no change in fasting and postprandial amylin levels in obese type 2 diabetic subjects up to 4 weeks after RYGB surgery as shown in Table S2 [73].

### 4.7. Insulin

Insulin is a 53 aminoacid hormone secreted by the beta cells of the pancreas. It increases uptake of glucose into target cells, promotes formation of glycogen, and decreases gluconeogenesis. A reduction in circulating insulin levels has been observed after RYGB surgery with improved insulin sensitivity as measured by homeostasis model assessment of insulin resistance (HOMA-IR). Improvement in insulin resistance has been reported as early as 6 days after RYGB surgery before any major weight loss has occurred [8]. The mechanism behind the early improvement in insulin resistance remains unclear. Caloric restriction early on after RYGB surgery can decrease hepatic glucose production [99] and reduce skeletal muscle insulin resistance [100]. In addition, the changes in adipocytokine and gut hormones profile that ensue following RYGB surgery act simultaneously to variable extents to improve insulin sensitivity.

## 5. Adipokines

Adipokines are bioactive peptides secreted from adipocytes that have multiple effects on metabolism with currently more than 50 adipokines identified [101]. The effect of these adipocyte-secreted factors on glucose homeostasis has been better elucidated in recent years. Both leptin, one of the first adipokines discovered to influence body fat mass and adiponectin, the most abundant adipocyte-derived protein, have been extensively studied in the regulation of carbohydrate and fat metabolism. Furthermore, favorable changes in their circulating levels after bariatric surgery have been assessed in various studies as described in the following paragraph. Other adipokines such as resistin, visfatin, vaspin, omentin, serum-retinol-binding protein (RBP)-4, chemerin, interleukin (IL)-6, plasminogen activator inhibitor (PAI-1), tumor necrosis factor (TNF), alpha, serum amyloid A, and angiotensinogen may have a role in obesity and T2D. However, data on changes postbariatric surgery are either minimal or nonexistent and were therefore not discussed in this paper.

### 5.1. Adiponectin

Adiponectin is a 244 aminoacid peptide. It is the most abundant adipokine secreted by the adipose tissue. Reduced levels of adiponectin are seen in obese patients [102]. Adiponectin levels are significantly lower as well in diabetic patients and in those with cardiovascular diseases compared to BMI-matched healthy controls [103, 104]. Studies in several adult populations have shown that adiponectin predicts the development of T2D [105–107]. Adiponectin circulates as three oligomeric isoforms: low molecular weight, medium molecular weight, and high molecular weight isoforms. The HMW adiponectin represents the major active form mediating the favorable metabolic effects of adiponectin [108]. Adiponectin regulates insulin sensitivity by increasing fatty acid oxidation, stimulating glucose uptake, and reducing hepatic gluconeogenesis [109]. An increase in adiponectin levels is observed with weight loss, and this increase is paralleled by an improved insulin resistance [110].

After RYGB surgery, an increase in adiponectin levels has been reported as shown in Table S3 [111–119].

### 5.2. Leptin

Leptin is a 167 aminoacid peptide secreted primarily by the adipose tissue and circulates at levels proportional to body fat. Leptin regulates appetite, energy expenditure, and body weight [120]. An increase in body fat is associated with an increase in leptin levels that act to decrease food intake. However, the elevated levels of leptin seen in obese individuals do not effectively suppress appetite because of an underlying resistance to the hormone [121]. Theories for leptin resistance suggest a defect in blood brain barrier transport of leptin induced by high-fat diets and abnormalities in leptin receptor signaling [122–124]. The effects of leptin on glucose homeostasis are still unclear. Leptin has been shown to enhance glucose uptake in skeletal muscles, reduce hepatic glucose output, increase fatty acid oxidation, and decrease insulin secretion by pancreatic beta cells [125].

Serum leptin levels have been shown to be reduced after RYGB surgery in several studies as presented in Table S4 [38, 57, 60, 110, 112, 115, 117, 126–130]. Whether bariatric surgery results in an improvement in the leptin-resistant state remains to be determined. However, the favorable changes in leptin and adiponectin levels after RYGB surgery are similarly seen with weight loss from other bariatric surgery procedures [131] as well as from pharmacological [132] and dietary methods [133, 134] suggesting that these changes are more related to fat loss rather than the RYGB surgery itself.

## 6. Discussion

**Figure 2:**
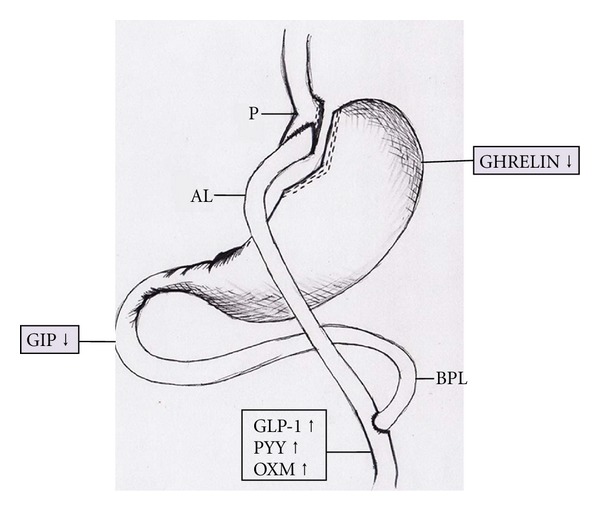
Changes in gut-related peptides post-RYGB surgery.

**Figure 3:**
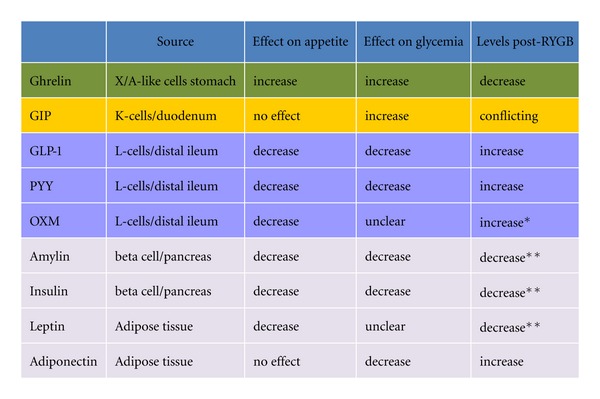
Summary of changes in peptides after RYGB surgery and their effects on glycemia and appetite. *Very few studies. **Hormonal levels decrease but glycemia improves due to improved sensitivity.

Studies evaluating hormonal changes after RYGB surgery have shown an overall positive change in hormones, favoring glycemic control. The orexigenic peptide ghrelin is reduced, while the anorexigenic GLP-1, oxyntomodulin, and PYY are increased. Hormones such as leptin, amylin, GIP, and insulin, to which a suggested state of resistance is observed in obesity and T2D tend to decrease, favor a restored homeostasis. Similarly, a change favoring improved insulin sensitivity with increased adiponectin is seen (Figure 2). A summary of the changes of all the peptides, along with their effect on glycemia and appetite is presented in Figure 3.

Despite the overall findings, there remain certain inconsistencies in the results which can be due to the following factors: some studies lacked an appropriate control group and merely assessed changes before and after RYGB surgery. In the few prospective controlled studies, the follow-up time did not exceed 2 years. As demonstrated in the Swedish Obese Subjects Study (SOS), short-term observations (<2 years) cannot mirror the long-term effects of bariatric surgery on comorbidities [135].

Sampling time points varied from one study to another. Some assessed fasting hormonal levels, whereas others measured the hormones in the postprandial state. Furthermore, a standardized meal test is lacking and assays used to measure the various hormones and peptides varied among different studies. For example, as previously mentioned ghrelin exists in two forms: acyl ghrelin that has been shown to produce stimulatory effects on food intake and desacyl ghrelin that induces a state of negative energy balance by inhibiting food intake and delaying gastric emptying [21]. Measuring total, acyl, or desacyl ghrelin will potentially give variable results. Therefore, this confounding factor must be taken into consideration. In addition, other potential explanations include the heterogeneity in the populations studied, variability in the method of reporting weight loss, and variability in the surgical techniques. Specific to changes relating to ghrelin, levels can vary depending on differences in the pouch size as well as configuration particularly if the pouch contains more fundic tissue. Variations in technique are widely noted among different surgeons. They are also noted within the same surgeon's experience as differences in patient's anatomy, body habitus, and effect of prior operations dictate modifications such as lengthening or shortening the pouch [7, 136]. The stoma diameter is important in determining how fast food is delivered to the small intestine and may play a role in the hormonal changes described above. Moreover, the length of the AL and BPL is not standardized and might have an impact on one or more of the gut incretins. Finally, one important factor not taken into account in most studies is the lack of standardized use of antidiabetic medications which can influence metabolites.

Even after consistent documentation of the hormonal changes, an important question remains in establishing the relationship of these alterations to metabolic control. Is the overall favorable hormonal milieu a result of the negative energy state or a causality of it? It may prove difficult to settle this point. However, controlled studies in the immediate postoperative phase, within one day to one week, would be helpful. Except for one study which measured ghrelin levels as early as two hours postoperatively [34], the vast majority obtain their first measurement two to three weeks postoperatively. Furthermore, comparison of changes following very low-caloric diets similar in intake to the immediate post-RYGB phase could prove interesting. These are lacking and in practice may be difficult to conduct.

## 7. Conclusion

In summary, in addition to the significant and sustained weight reduction achieved by RYGB surgery, improvement in obesity comorbidities, insulin resistance, and glycemic control is noted. This amelioration is attributed, at least partly, to an alteration in gut peptide release and adipokines. The timing and exact changes of these hormones, as well as their etiologic link to metabolic control postsurgery need to be better established.

Thus, long-term controlled studies and additional research focusing on the very early phase post-RYGB are required for a comprehensive appraisal of the mechanisms behind T2D and its control. These advances will help identify new targets for pharmacological treatment of diabetes.

## Supplementary Material

We have provided 3 supplementary tables (Table S1, S3, and S4) illustrating the changes in the following hormones PYY, adiponectin and leptin after RYGB surgery that are consistent among the various studies. Table S2 shows the only two studies looking at the changes in amylin after RYGB surgery.

## Figures and Tables

**Table 1:** Ghrelin and RYGB surgery.

Author/year	Type of study	Subjects	Preop BMI	% weight loss	F/U time	Change in hormone
Geloneze et al. 2003 [33]	Prospective controlled	28 RYGB surgery 14 T2D 14 NGT lean group	56.3 ± 10.2 24.2 ± 1.5	% EWL 67.4 ± 13.4	1 y	Lower ghrelin levels in obese compared to lean presurgery; No difference in fasting ghrelin in T2D and NGT before surgery; Decrease in fasting ghrelin in both T2D and NGT after surgery.
Lin et al. 2004 [32]	Prospective controlled	34 RYGB 4 VBG 4 ARS 4 lean ARS	47.0 ± 0.7 43.7 ± 2.5 40.0 ± 2.0 23.8 ± 0.9	NA	30 min postop	Ghrelin higher in lean ARS compared to pre-RYGB; Decrease in ghrelin levels post-RYGB to levels lower that lean ARS;
Frühbeck et al. 2004 [37]	Prospective controlled	8 RYGB 8 AGB 8 Conv 6 Total gastrectomy	44.2 ± 2.6 44.8 ± 1.6 43.7 ± 1.5 29.9 + 1.1	NA	6 mo	At 6 mo, lower fasting ghrelin in RYGB and gastrectomy groups compared to AGB and conv group; No differences in fasting ghrelin at 6 mos between RYGB group and gastrectomy group.
Couce et al., 2006 [34]	Prospective controlled	49 obese (30 F) RYGB 19 obese (9 F) other GI surgeries	50 ± 5.3 29.8 ± 3.1	NA	2 hr 10 d 6 mo	Decrease in fasting ghrelin at 2 hr in both groups compared to preop; Decrease in fasting ghrelin 10 d postop in only RYGB group compared to preop; At 6 mo, no change in ghrelin levels in both groups compared to preoperative levels.
Morínigo et al. 2008 [35]	Prospective controlled	25 non diabetics RYGB (6 F) 6 nonobese (2 F) 10 severely obese T2D (5 F) RYGB	48.8 ± 1.2 49.2 ± 2.0	43.0 ± 2.3	6 and 52 wk	Decrease in fasting plasma ghrelin at 6 wk postop; At 52 wk, plasma ghrelin returned to baseline levels.
Karamanakos et al. 2008 [40]	Prospective controlled	16 RYGB (12 F, 2 T2D) 16 LSG (15 F, 1 IGT)	46.6 ± 3.7 45.1 ± 3.6	% EWL^a^ 60.5 ± 10.7 69.7 ± 14.6	1, 3, 6 and 12 mo	No significant change in fasting ghrelin RYGB group; Significant decrease in LSG.
Oliván et al. 2009 [39]	Prospective controlled	21 T2D 10 RYGB 11 diet 9 obese nondiabetics	47.4 ± 10.6 42.8 ± 3.8 45.5 ± 7.1	NA	10 Kg weight loss	No change in fasting ghrelin after RYGB
Frühbeck et al. 2004 [38]	Retrospective controlled	6 RYGB 7 AGB 3 BPD	42.6 ± 1.6 45.6 ± 1.8 60.5 ± 7.3	50.1 ± 4.4 42.2 ± 3.1 54.2 ± 4.3	6.1 ± 0.4 mo 7.0 ± 0.6 mo 4.4 ± 0.8 mo	Significant decrease in fasting ghrelin in RYGB group compared to the other 2 groups
Foschi et al. 2008 [36]	Retrospective controlled	10 RYGB (9 F) 12 VBG (11 F)	44.1 ± 1.8 42.9 ± 1.6	20	20% reduction in BMI ( = 131 ± 6 d for RYGB) (119 ± 4.2 for VBG)	Basal ghrelin plasma levels reduced after RYGBP but increased after VBG
Rodieux et al. 2008 [41]	Cross-sectional controlled	8 RYGB 6 GB 8 weight matched	44.9 ± 1.8 41.1 ± 0.5 29.2 ± 0.8	47.8 ± 3.3 32.4 ± 2.0	9 to 48 mo 25 to 85 mo	No change in fasting ghrelin Maximal PP suppression of ghrelin greatest in RYGB group

Abbreviations: ABG: adjustable gastric banding, ARS: anti-reflux surgery, Conv: conventional weight loss, GB: gastric banding, GI: gastrointestinal, IGT: impaired glucose tolerance, NA: data not available, LSG: laparoscopic sleeve gastrectomy, Postop: postoperatively, RYGB: Roux-en-y gastric bypass, T2D: type 2 diabetes, VBG: vertical banded gastroplasty.

^a^% EWL: excess weight loss = [(operative weight − follow-up weight)/operative excess weight] × 100.

**Table 2:** GLP-1 and RYGB surgery.

Author/year	Type of study	Subjects	Preop BMI	% weight loss	F/U time	Change in hormone
Morínigo et al. 2006 [51]	Prospective controlled	9 (7 F) RYGB non diabetic 6 obese (4 F)	47.4 ± 6.1 43.6 ± 7.9	NA	6 wk	Greater increase in active GLP-1 postmeal in RYGB group postop compared to weight-matched obese
Laferrère et al. 2007 [50]	Prospective controlled	8F T2DM RYGB 7 non diabetic obese	43.6 ± 6.8 37.1 ± 11.6	NA	1 mo	Fasting- and glucose-stimulated GLP-1 similar in S and C 1 month after RYGB, increase in GLP-1 (total and active) in response to oral glucose
Reinehr et al. 2007 [59]	Prospective controlled	30 obese (26 F) 19 RYGB 11 GB	45.7 ± 7.4	50%	2 y	Decrease in fasting GLP-1 in both groups
Le Roux et al. 2007 [56]	Double-blind randomized prospective controlled	7 RYGB 6 AGB	44.5 ± 2.9 41.9 ± 7.5	NA	9.5 ± 1.5 mo 17 ± 1.4 mo	Early (2 d) and increased responses of PP GLP-1 in RYGB group only
Laferrère et al. 2008 [49]	Prospective controlled	9 F T2D RYGB 10 F T2D diet induced weight loss	43.3 ± 6.2 43.3 ± 3.6	NA	1 mo 10 Kg weight loss	Increase in total GLP-1 after oral glucose and GLP-1 AUC after RYGB but not after diet
Peterli et al. 2009 [54]	Randomized prospective controlled	13 RYGB 14 LSG	47 ± 6.4 45.7 ± 6.7	NA	1 wk and 3 mo	Increased PP GLP-1 RYGB > LSG
Clements et al. 2004 [61]	Prospective uncontrolled	20 obese (15 F) with T2D	52.7 ± 8.8	NA	2, 6, and 12 wk postop	No change in fasting GLP-1 at any time point
Rubino et al. 2004 [60]	Prospective uncontrolled	S: 10 (9 F, 6 T2D) obese RYGB	46.2	NA	3 wk	No change in fasting GLP-1 in postop
Borg et al. 2006 [57]	Prospective uncontrolled	6 RYGB	48.3	NA	1, 3, 6 mo postop	PP GLP-1 AUC increased at 6 mo postop
Morínigo et al. 2006 [52]	Prospective uncontrolled	34 RYGB (23 F, 12 NGT, 12 IGT, 10 T2D)	49.1 ± 1.0	NGT: 34.5 ± 1.4 IGT: 29.2 ± 1.9 DM: 32.0 ± 2.4	6 wk 12 mo	Increase in PP GLP-1 AUC response in IGT and NGT at 6 wk Increase in PP GLP-1 AUC response in all 3 groups at 12 mo
De Carvalho et al. 2009 [53]	Prospective uncontrolled	11 NGT (9 F) RYGB 8 AGM (4 T2DM, 4 IGT) (7 F) RYGB	46.1 ± 2.27 46.5 ± 2.04%	39.3 ± 2.24 36.4 ± 2.6	T1: First evaluation T2: presurgery T3: 9 mo after surgery	Increase in GLP-1 levels after OGTT in both groups at T3
Kashyap et al., 2010 [73]	Prospective uncontrolled	16 (7 F) T2D 9 RYGB 7 GR	47 ± 9	10%	4 wk	No change in fasting GLP-1 in both groups Increase in PP GLP-1 response in RYGB group only
Le Roux et al. 2006 [55]	Cross-sectional controlled	6 RYGB 6 GB 12 obese 15 lean	49.8 46.1 47.1 23.8	NA	6 to 36 mo	Higher postprandial GLP-1 response in RYGB group compared to fasting levels and to other groups
Korner et al. 2007 [58]	Cross-sectional controlled	13 F non diabetic RYGB 10 F BND 13 F OW	31.3 ± 1.3 36.1 ± 1.7 36.1 ± 2.2	35.6 ± 2.4 24.6 ± 2.3	24.6 ± 2 mo postop	Fasting GLP-1 similar in all groups At 30 min postmeal, GLP-1 higher in RYGB group compared to BND and OW GLP-1 AUC at 180 min greater in RYBG group compared to other groups
Rodieux et al. 2008 [41]	Cross-sectional controlled	8 RYGB 6 GB 8 weight matched	44.9 ± 1.8 41.1 ± 0.5 29.2 ± 0.8	47.8 ± 3.3 32.4 ± 2.0	9 to 48 mo (RYGB) 25 to 85 mo (GB)	No difference in fasting GLP-1 between 3 groups; Exaggerated GLP-1 PP Response in RYGB.

Abbreviations: AUC: area under the curve, AGM: abnormal glucose metabolism, BND: adjustable gastric banding, GR: gastric restrictive, IGT: impaired glucose tolerance, LSG: laparoscopic sleeve gastrectomy, NA: data not available, NGT: normal glucose tolerance, OGTT: oral glucose tolerance test, OW: overweight, Postop: postopertaively, PP: postprandial, RYGB: Roux-en-y gastricbBypass, T2D: type 2 diabetes.

**Table 3:** GIP and RYGB surgery.

Author/year	Type of study	Subjects	Preop BMI	% weight loss	F/U time	Change in hormone
Laferrère et al. 2007 [50]	Prospective controlled	8 F T2D RYGB 7 nondiabetic obese	43.6 ± 6.8 37.1 ± 11.6	NA	1 mo	Fasting- and glucose-stimulated GIP similar in S and C 1 month after RYGB, increase in GIP in response to oral glucose.
Laferrère et al. 2008 [49]	Prospective controlled	9 F T2D RYGB 10 F T2D diet-induced weight loss	43.3 ± 6.2 43.3 ± 3.6	NA	1 mo 10 Kg weight loss	No change in fasting GIP in both groups. Increase in peak GIP after OGTT in RYGB group only.
Rubino et al., 2004 [60]	Prospective uncontrolled	10 (9 F, 6 T2D) obese RYGB	46.2	NA	3 wk	Baseline GIP higher in diabetics compared to nondiabetics. Decrease in fasting GIP in diabetics only.
Clements et al. 2004 [61]	Prospective uncontrolled	20 obese (15 F) with T2D	52.7 ± 8.8	NA	2, 6 and 12 wk postop	Decrease in fasting GIP at 6 and 12 wk.
Whitson et al. 2007 [72]	Prospective uncontrolled	10 (9 F, 5 T2D) RYGB	50 ± 6	NA	6 mo	No change in GIP postop (nonfasting).
Kashyap et al. 2010 [73]	Prospective uncontrolled	16 (7 females) T2D 9 RYGB 7 GR	47 ± 9	10%	4 wk	No change in fasting or PP GIP in both groups.
Korner et al. 2007 [58]	Cross-sectional controlled	13 F RYGB 10 F BND 13 F overweight	31.3 ± 1.3 36.1 ± 1.7 36.1 ± 2.2	35.6 ± 2.4 24.6 ± 2.3	24.6 ± 2 mo postop	Blunted PP GIP peak after RYGB

Abbreviations: BND: adjustable gastric banding, GR: gastric restrictive, NA: data not available, OGTT: oral glucose tolerance test, Postop: postoperatively, PP: postprandial, RYGB: Roux-en-y gastric bypass, T2D: type 2 diabetes.

## Abbreviations

AL: Alimentary limb
BPL: Biliopancreatic limb
DPPIV: Dipeptidyl peptidase IV
GH: Growth hormone
GHSR1: Growth hormone secretagogue receptor 1
GIP: Gastric inhibitory polypeptide
GLP-1: Glucagon-like peptide-1
NPY2: Neuropeptide Y subtype 2
OXM: Oxyntomodulin
PAI-1: Plasminogen activator inhibitor
PYY: Peptide YY
RBP-4: Retinol-binding protein 4
RYGB: Roux-en-y gastric bypass
T2D: Type 2 diabetes.
