# Supplementary material for: Role of Gut-Related Peptides and Other Hormones in the Amelioration of Type 2 Diabetes after Roux-en-Y Gastric Bypass Surgery
Source: ISRN Endocrinol. 2012 May 7;2012:504756. doi: 10.5402/2012/504756 (PMC3353119; doi:10.5402/2012/504756)
Supplement: Supplementary file 1 — We have provided 3 supplementary tables (Table S1, S3, and S4) illustrating the changes in the following hormones PYY, adiponectin and leptin after RYGB surgery that are consistent among the various studies. Table S2 shows the only two studies looking at the changes in amylin after RYGB surgery. [file 504756.f1.pdf]

Table S1: PYY and RYGB Surgery

Author/year	Type of Study	Subjects	Preop BMI	% weight loss	F/U time	Change in Hormone
**Morinigo 2006 [51]**	Prospective controlled	9 (7 F) RYGB<br>6 obese (4 F)	47.4 ± 6.1<br>43.6 ± 7.9	NA	6 wk	Increase in PP total PYY AUC response in RYGB group (greater than in obese controls)
**Reinehr 2007[59]**	Prospective controlled	30 obese adults (26 F)<br>19 RYGB<br>11 GB	45.7±7.4	50	2 y	Increase in fasting PYY in both groups
**Le Roux 2007 [56]**	Double blind randomized prospective controlled	7 RYGB<br>6 AGB	44.5 ± 2.9<br>41.9 ± 7.5	NA	9.5 ±1.5 mo<br>17 ± 1.4 mo	Early (2 days) and increased responses of PP PYY in RYGB group only
**Karamanakos 2008[40]**	Prospective controlled	16 RYGB (12 F, 2 T2D)<br><br>16 LSG (15 F, 1 glucose intolerance)	46.6 ± 3.7<br><br>45.1 ± 3.6	% EWL<br>60.5 ± 10.7<br><br>69.7 ± 14.6	1, 3,6 and 12 mo	Fasting PYY increased significantly in both groups
**Morinigo 2008[35]**	Prospective controlled	25 non diabetics RYGB (6F)<br><br>6 non obese (2F)<br><br>10 severely obese T2D (5F) RYGB	48.8 ± 1.2<br><br>49.2 ± 2.0	43.0 ± 2.3	6 and 52 wk	No change in fasting PYY at 6 weeks<br>Increase in PP PYY AUC response at 6 and 52 wk in RYGB group (significantly greater compared to normal weight controls)
**Olivan 2009 [39]**	Prospective controlled	21 T2D<br>10 RYGB<br>11 diet<br><br>9 obese non diabetics	 47.4 ± 10.6<br>42.8 ± 3.8<br><br>45.5 ± 7.1	NA	After a 10 Kg weight loss	Increase in PP PYY 3-36 AUC in surgical group
**Valderas 2010 [89]**	Prospective controlled	8 RYGB<br>8 SG<br>8 diet<br>8 lean	37.8 ± 0.8<br>35.3 ± 0.7<br>39.1 ±1.7<br><br>21.7 ± 0.7	17.7 ±3<br>14.9 ± 2.4<br>16.6 ± 4	2 mo	PYY AUC increased in RYGB and SG group but not in diet group

**Borg 2006 [57]**	Prospective uncontrolled	6 RYGB	48·3	NA	1,3,6 mo post op	PP PYY increased at 3 and 6 mo post op
**Le Roux 2006 [55]**	Cross-sectional controlled	6 RYGB<br>6 GB<br>12 obese<br>15 lean	49.8<br>46.1<br>47.1<br>23.8	NA	6 to 36 mo	Higher postprandial PYY response in RYGB group compared to fasting levels and to other groups
**Rodieux 2008 [41]**	Cross-sectional controlled	8 RYGB<br>6 GB<br>8 weight matched	44.9 ± 1.8<br>41.1 ± 0.5<br>29.2 ± 0.8	NA	9 to 48 mo (RYGB)<br>25 to 85 mo (GB)	Fasting PYY and PYY response to oral glucose significantly greater in RYGB group compared to other groups

Abbreviations: AGB: adjustable gastric banding, LSG: laparoscopic sleeve gastrectomy, NA: data not available, Post op: postoperatively, PP: post prandial, RYGB: Roux-en-y gastric bypass, T2D: type 2 diabetes.

Table S2: Amylin and RYGB Surgery

Author/year	Type of Study	Subjects	Preop BMI	% weight loss	F/U time	Change in Hormone
**Bose 2010 [99]**	Prospective controlled	11(F) RYGB T2D<br>8 (F) non diabetic obese	45.1 ± 8.2<br>45.1 ± 7.5	NA	1, 6,12 mo	Fasting amylin and PP amylin AUC decrease at 1 month and remain unchanged up to 1 year
**Kashyap 2010 [73]**	Prospective uncontrolled	16 (7 F) T2D<br>9 RYGB<br>7 GR	47±9	10	4 wks	No change in fasting or PP amylin in both groups

Abbreviations: AUC: area under the curve, NA: data not available, PP: post prandial, RYGB: Roux-en-y gastric bypass, T2D: type 2 diabetes

Table S3: Adiponectin and RYGB Surgery

Author/year	Type of Study	Subjects	Preop BMI	% weight loss	F/U time	Change in Hormone
**Serra 2006 [112]**	Prospective controlled	70 (41 F) RYGB<br>24 (15 F) normal weight	53.3 ±9.6<br>23.5 ±2.6	NA	12 mo	Increase in adiponectin in the RYGB group compared to baseline and value reached higher than control group
**Whitson 2007[113]**	Prospective controlled non randomized	10 (9 F)<br>5 diabetics<br>5 nondiabetics	50 ± 6	NA	6 mo	Increase in adiponectin at 6 mo in both diabetics and non diabetics more so in nondiabetics

**Garcia de la Torre 2008 [114]**	Prospective controlled	45 obese F<br>VBG =17<br>GB=17<br>BPD=11<br><br>9 lean	49.9±8.1	33.6	9-12 mo	Adiponectin lower in obese compared to lean group.<br>Increase in adiponectin in all groups after surgery
**Trakhtenbroit 2009 [115]**	Prospective controlled	10 F RYGB<br><br>5 F AGB	47.6 ±2.2<br><br>44.93 ± 3.51	37.36 ± 3.13<br><br>19.54 ± 3.81	3 mo<br>9 mo<br>24 mo	Increase in adiponectin levels more in the RYGB group
**Holdstock 2003 [116]**	Prospective uncontrolled	66 (54 F)	44.8 ±6.4	22.3	6 mo<br>12 mo	Increase in adiponectin at 6 and 12 mo
**Swarbrick 2006 [117]**	Prospective uncontrolled	19 F	45.6 ±1.6	31.8	1 mo<br>12 mo	No change at one month<br>Increase in HMW and total adiponectin at 12 mo
**Lin 2007 [118]**	Prospective uncontrolled	28 F	48.2 ±0.7	27	1 mo<br>6 mo	Increase in adiponectin at 1 and 6 months
**Vilarrasa 2007 [119]**	Prospective uncontrolled	65 (60 F)	49·2 ± 7·7	35·4	12 mo	Increase in adiponectin after surgery
**Coughlin 2007 [120]**	Prospective uncontrolled	6 F	57.1 ± 4.1	36 ± 5	12 mo	Increase in adiponectin

Abbreviations: ABG: adjustable gastric banding, BPD: biliopancreatic diversion, GB: gastric banding, NA: data not available, RYGB: Roux-en-Y gastric bypass, VBG: vertical banded gastroplasty

Table S4: Leptin and RYGB Surgery

Author/year	Type of Study	Subjects	Preop BMI	% weight loss	F/U time	Change in Hormone
**Molina 2003 [127]**	Prospective controlled	29 F RYGB (9 T2D)<br><br>13 F non obese	49 ± 6	NA	1,3,6 mo post op	Leptin decreased at all time points
**Fruhbeck 2004 [38]**	Prospective controlled	16 (7 F)<br>6 RYGB<br>7 AGB<br>3 BPD	 42.6 ± 1.6<br>45.6 ± 1.8<br>60.5 ± 7.3	 50.1 ± 4.4<br>42.2 ± 3.1<br>54.2 ± 4.3	 6.1 ± 0.4 mo	Decrease in leptin post op in all 3 groups

**Bobbioni-Harsch 2000 [128]**	Prospective uncontrolled	20 F RYGB	43.9 ± 1.3	NA	3,6,12 mo post op	Leptin decreased post op
**Holdstock 2003 [116]**	Prospective uncontrolled	66 obese (54 F) (2 on medical trt for T2D)	45	30%	6 and 12 mo	Decrease in leptin levels at 6 and 12 mo
**Faraj 2003 [111]**	Prospective uncontrolled	50 RYGB (39 F) (10 receiving OHA not specified)	48.5 ± 10.1	36.4 ± 9.6	15 ± 6 mo	Decrease in leptin levels
**Das 2003 [129]**	Prospective uncontrolled	30 (24 F) RGYB (diabetics excluded)	50.1 ± 9.3	38 ± 19	1 y	Decrease in fasting leptin levels
**Rubino 2004 [60]**	Prospective uncontrolled	S: 10 (9 F, 6 T2D) obese RYGB	46.2	NA	3 wk	Decrease in fasting leptin in all
**Borg 2006 [57]**	Prospective uncontrolled	6 RYGB	48·3	NA	1,3,6 mo post op	Fasting leptin decreased at 3 and 6 mo post op
**Lin 2007 [118]**	Prospective uncontrolled	28 F	48.2 ±0.7	27	1 mo<br>6 mo	Decrease in leptin at 1 and 6 mo
**Czupryniak 2007 [130]**	Prospective uncontrolled	68 (39 F) RYGB	44.4±6.8	38.0±9.0 (max weight reduction at 15 ± 4 months)	Q 1-2 mo during 1st y<br>Q 3 mo during 2nd-3rd y	At time of max weight loss(15 ±4 mo) ,leptin decreased significantly
**Whitson 2007 [113]**	Prospective uncontrolled	10 (9 F, 5 T2DM) RYGB	50 ± 6	NA	6 mo	Decrease in leptin in both diabetics and nondiabetics
**Swarbrick 2008 [131]**	Prospective uncontrolled	19 F RYGB	45.6 ± 1.6	49.6± 2.5	1, 3, 6,12 mo post op	Fasting leptin significantly lower than baseline at all time points

Abbreviations: ABG: adjustable gastric banding, BPD: biliopancreatic diversion, NA: data not available, OHA: oral hypoglycemic agents, Post op: postoperatively, RYGB: Roux-en-y gastric bypass, T2D: type 2 diabetes, trt: treatment.
